# Case Report: Soft-tissue-preserving fixation for displaced segmental maxillary fractures involving partially detached bone segments

**DOI:** 10.3389/froh.2026.1889166

**Published:** 2026-08-18

**Authors:** Xin Jin, Yu Zhuang, Ziqing He, Jianfei Zhang, Jun Shi, Wenbin Zhang

**Affiliations:** 1 Department of Oral and Craniomaxillofacial Surgery, Shanghai Ninth People’s Hospital, Shanghai Jiao Tong University School of Medicine, Shanghai, China; 2 College of Stomatology, Shanghai Jiao Tong University, Shanghai, China; 3 National Center for Stomatology, Shanghai, China; 4 National Clinical Research Center for Oral Diseases, Shanghai, China; 5 Shanghai Key Laboratory of Stomatology, Shanghai, China; 6 Shanghai Research Institute of Stomatology, Shanghai, China; 7 School of Stomatology, Inner Mongolia Medical University, Hohhot, Inner Mongolia, China

**Keywords:** dentoalveolar segment, epimucosal fixation, segmental maxillary fracture, soft-tissue preservation, transmucosal screw fixation

## Abstract

**Background:**

Displaced segmental maxillary fractures involving partially detached bone segments are difficult to manage because treatment requires both stable fixation and preservation of residual vascularity. Excessive mucoperiosteal elevation may further compromise the blood supply of a displaced segment and increase the risk of bone resorption, sequestration, or necrosis. This report presents two cases of displaced segmental maxillary fractures treated with soft-tissue-preserving fixation strategies.

**Case presentation:**

Two patients with displaced segmental maxillary fractures were managed using individualized mucosa-related fixation techniques. In the first case, a 35-year-old man presented with a large displaced anterior maxillary segment and loss of local buttress support. After restoration of the occlusal relationship, the segment was stabilized using intraoral epimucosal reconstruction plate fixation. In the second case, a 51-year-old woman sustained an open maxillofacial injury with a dentoalveolar segment involving teeth 23–25. The segment showed complete loss of bony continuity with the surrounding maxilla, with only limited labial mucosal attachment remaining. Palatal transmucosal screw fixation with long titanium screws was performed to stabilize the segment while preserving the residual labial soft-tissue attachment. Both patients achieved stable reduction and satisfactory healing. No obvious postoperative necrosis, sequestration, infection, or exposure of fixation materials was observed. In the second case, the preserved dentoalveolar segment achieved bony healing, and teeth 23–25 remained stable during follow-up.

**Conclusion:**

In these two cases of displaced segmental maxillary fractures, preservation of residual soft-tissue attachments appeared to contribute to segment survival when adequate reduction and stable fixation could be achieved. Mucosa-related fixation may be considered as an individualized option in carefully selected patients with potentially viable partially detached segments. These observations are preliminary and hypothesis-generating, and further studies with larger case series and longer follow-up are needed to clarify the indications, limitations, and long-term outcomes of this approach.

## Introduction

Maxillary fractures associated with displaced or partially detached bone segments represent one of the more challenging injury patterns in oral and maxillofacial trauma. These injuries often result from high-energy trauma and are frequently accompanied by disruption of the midfacial buttresses, dentoalveolar injury, occlusal derangement, facial deformity, and soft-tissue compromise (1, 2). The primary goals of treatment include restoration of midfacial skeletal support, re-establishment of occlusion, anatomical reduction of displaced fragments, and stable fixation to prevent secondary displacement, malunion, infection, or delayed healing (3, 4).

Open reduction and internal fixation remain the standard approach for most displaced maxillofacial fractures, as rigid or functionally stable fixation provides reliable maintenance of bone segment position and facilitates early functional recovery (3, 5). However, in selected maxillary fractures with segmental displacement or near-detachment of bone fragments, conventional plate fixation after wide flap elevation may not always be the optimal strategy. When a displaced bone segment retains only limited mucosal, periosteal, or soft-tissue attachments, its postoperative viability may depend largely on the preservation of residual vascularity (6). Additional periosteal stripping and extensive exposure, although helpful for direct visualization and plate adaptation, may further compromise the already vulnerable blood supply and increase the risk of bone resorption, sequestration, infection, or segmental necrosis (7, 8).

Therefore, the management of such fractures requires a careful balance between mechanical stability and biological preservation. In selected cases, mucosa-related fixation techniques have been described as alternative strategies that may provide sufficient stabilization while avoiding extensive mucoperiosteal elevation and minimizing further disruption of residual soft-tissue attachments (7, 9). These techniques may be particularly useful when the displaced segment can be reduced under limited exposure, when the remaining soft-tissue attachment is considered critical for segment survival, or when further flap elevation may increase the risk of devascularization (10–13).

In this article, we report two cases of displaced segmental maxillary fractures treated with fixation strategies designed to preserve residual soft-tissue attachments. In the first case, intraoral epimucosal reconstruction plate fixation was used to stabilize a large displaced anterior maxillary segment. In the second case, palatal transmucosal screw fixation with long titanium screws was performed to preserve a detached dentoalveolar segment with limited labial mucosal attachment. Both segments survived without obvious postoperative necrosis or sequestration. Through these two cases and a review of the relevant literature, the objective of this report is to describe our clinical experience with two soft-tissue-preserving fixation methods for selected displaced segmental maxillary fractures and to discuss the clinical considerations that may support attempted preservation of a partially detached segment in carefully selected patients.

## Case report 1

**Figure 1:**
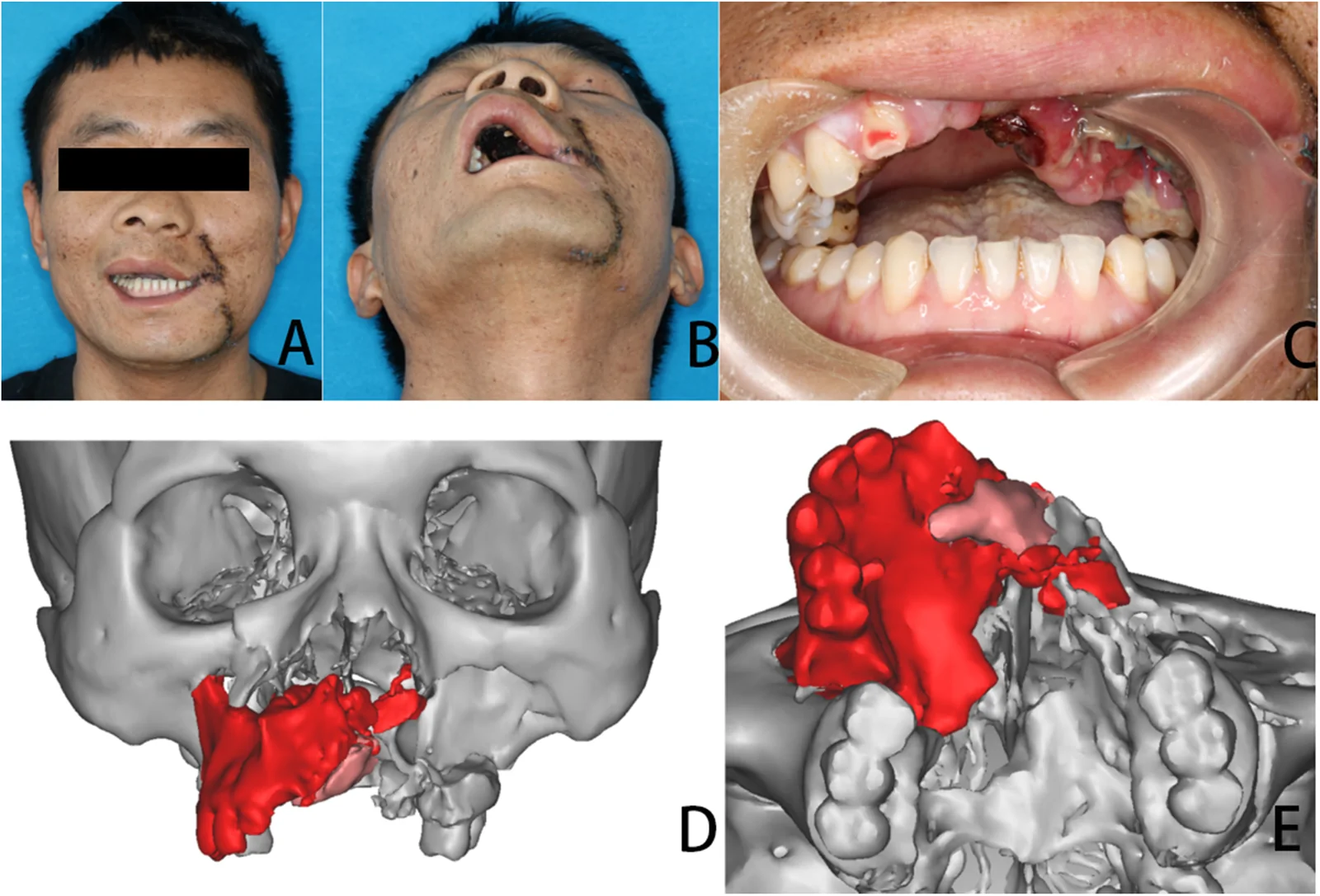
Preoperative clinical and radiographic images of case 1. **(A,B)** Extraoral photographs. **(C)** Intraoral photograph. **(D,E)** Three-dimensional CT reconstructions.

A 35-year-old man was admitted to our department 10 days after sustaining a blunt maxillofacial injury at a construction site. The patient had no significant past medical history and reported no history of diabetes, hypertension, smoking-related systemic disease, or medication use that might adversely affect wound healing. No known drug allergies were reported, and his general condition on admission was stable. On admission, he presented with marked facial asymmetry and bilateral midfacial swelling. Previously sutured facial and chin wounds were observed, with crust formation but without obvious erythema, swelling, or purulent discharge. The patient complained of numbness in the left mental region. Preoperative clinical photographs showed midfacial swelling, facial asymmetry, soft-tissue wounds, and intraoral traumatic changes (Figures 1A–C).

Intraoral examination revealed retained sutures, malocclusion, bilateral premature posterior dental contact, and a maximal mouth opening of approximately two finger breadths. A crown fracture of tooth 11 was noted, and teeth 21, 22, 24, and 25 were missing. Palatal sutures were also present. No obvious tenderness was detected in either temporomandibular joint region.

Computed tomography and three-dimensional reconstruction demonstrated multiple fractures involving the bilateral maxillae, maxillary sinus walls, and nasal bones, accompanied by marked swelling of the surrounding soft tissues and mucosal thickening of both maxillary sinuses. The anterior maxilla and alveolar process were severely involved. In particular, the right anterior maxillary segment was partially detached and markedly displaced, with substantial disruption of local skeletal support (Figures 1D,E).

Because the displaced segment retained residual soft-tissue attachments, its viability was considered to depend largely on preservation of residual vascularity. Wide flap elevation and conventional plate fixation directly on the bone surface might have caused further devascularization and increased the risk of postoperative bone resorption, sequestration, or segmental necrosis. Given the relatively large size of the detached segment and the comminuted fracture pattern, conventional miniplate fixation under limited exposure was considered less suitable to provide both mechanical stability and biological preservation. Therefore, intraoral epimucosal fixation using a 2.0-mm locking reconstruction plate system (DePuy Synthes, West Chester, PA, USA) and 2.0-mm titanium screws, each 6 mm in length, was selected to stabilize the segment while minimizing additional mucoperiosteal stripping.

The operation was performed under general anesthesia with the patient in the supine position. After routine disinfection and draping, intraoral disinfection was performed. Seven traction screws were placed to assist fracture reduction and traction. The intraoral wound was thoroughly debrided, the torn gingiva in the maxillary vestibular region was repositioned and sutured, and granulation tissue in the fracture area was removed.

Intermaxillary wiring was applied to guide occlusal restoration. The displaced bone segment was adjusted until a stable occlusal relationship was achieved. Subsequently, a reconstruction plate was positioned in the maxillary vestibular region and fixed with titanium screws. In total, one reconstruction plate and nine screws were used. After fixation, the intermaxillary wiring was released, and the occlusion was reassessed and confirmed to be stable.

The surgical field was thoroughly irrigated. Local soft-tissue advancement was performed to cover the wound and obliterate the dead space. The wound was then closed carefully. Postoperatively, elastic traction was applied to maintain the occlusal relationship and was adjusted according to occlusal stability during the early postoperative period. The patient received routine postoperative antibiotics, analgesic treatment, and oral hygiene care. Chlorhexidine mouth rinses were recommended, and the patient was instructed to clean carefully around the exposed epimucosal plate and avoid direct mechanical irritation to the fixation area. A liquid or soft diet was recommended during the early healing period. Follow-up examinations focused on occlusion, mucosal healing, plaque accumulation around the exposed hardware, wound dehiscence, infection, and plate exposure. The patient returned to the ward after recovery from anesthesia.

**Figure 2:**
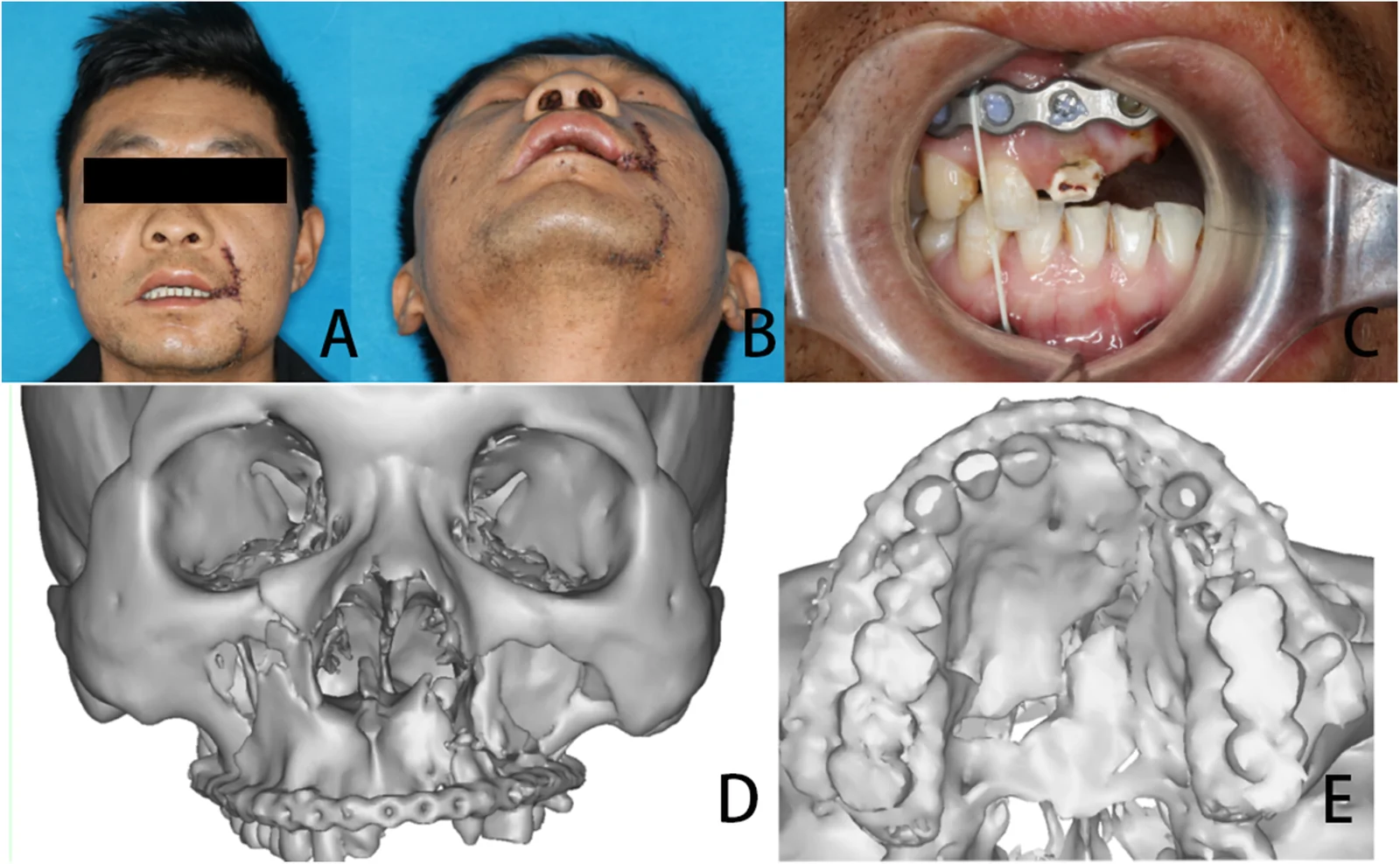
Postoperative clinical and radiographic images of case 1. **(A,B)** Extraoral photographs. **(C)** Intraoral photograph. **(D,E)** Three-dimensional CT reconstructions.

The patient recovered uneventfully after surgery. His facial contour improved markedly, and both occlusion and masticatory function recovered satisfactorily. Postoperative three-dimensional CT reconstruction showed satisfactory reduction of the displaced maxillary segment and stable fixation with the intraoral epimucosal reconstruction plate (Figure 2).

During follow-up, the intraoral mucosa healed well, and no obvious swelling, wound dehiscence, infection, or reconstruction plate exposure was observed. Four months after the initial surgery, the reconstruction plate was removed. At the same time, bone graft material and membrane were placed in the alveolar defect region of teeth 21 and 22, and autogenous bone harvested from the left mandibular body was grafted into the alveolar defect in the region of teeth 24 and 25. Three months after plate removal and bone grafting, follow-up examination showed satisfactory survival of the previously detached maxillary segment, with no obvious evidence of bone necrosis or sequestration.

## Case report 2

A 51-year-old woman was admitted to our department more than 5 h after sustaining an open maxillofacial injury caused by an unmanned aerial vehicle propeller. On admission, the patient had no significant past medical history relevant to wound healing or fracture management. She was conscious and hemodynamically stable on admission, and no major systemic comorbidity or drug allergy was reported. A transverse facial laceration extending from the right buccal region to the left infra-auricular region was observed, measuring approximately 15 cm in length. The wound margins were irregular, with active bleeding. The injury extended into the muscular layer and communicated with the oral cavity. Exposed maxillary bone, fractured bone fragments, broken teeth, and partial loss of the maxillary dentition were observed within the wound.

**Figure 3:**
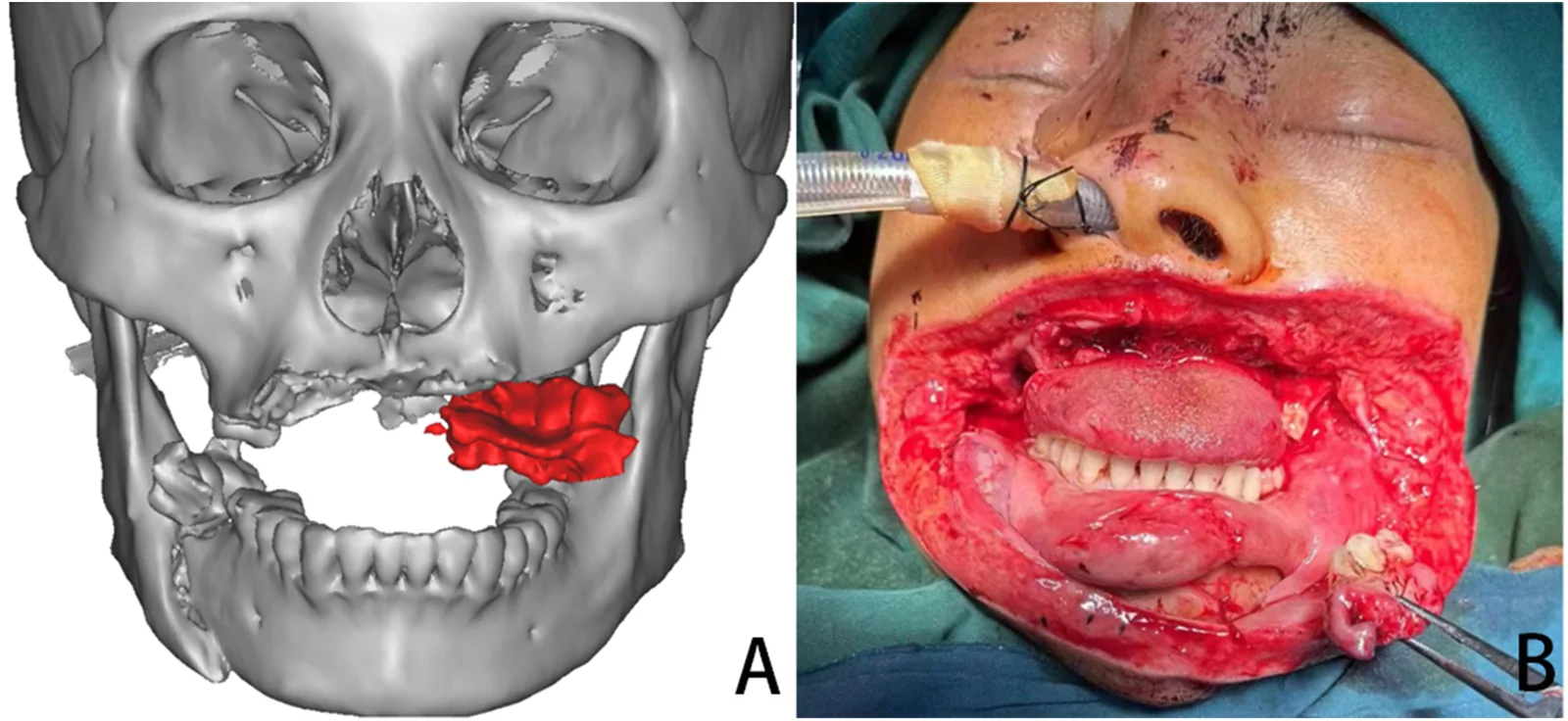
Preoperative and intraoperative images of case 2. **(A)** Preoperative three-dimensional CT reconstruction. **(B)** Intraoperative photograph.

Computed tomography and three-dimensional reconstruction revealed multiple fractures involving both the maxilla and mandible. The maxillary fractures involved the anterior maxilla, alveolar process, and palate, with comminution, displacement, and segmental bone loss. A linear fracture was also observed in the right mandibular region around tooth 46, with obvious displacement of the fracture ends and lateral displacement of the local bone segment (Figure 3A).

Intraoperatively, the dentoalveolar segment involving teeth 23, 24, and 25 showed complete loss of bony continuity with the surrounding maxilla, with only limited labial mucosal attachment remaining (Figure 3B). This finding suggested that the viability of the segment might largely depend on residual vascularity provided by the remaining soft-tissue attachment. Further elevation of the labial or palatal mucoperiosteum for conventional plate fixation could have caused additional devascularization, thereby increasing the risk of bone resorption, necrosis, or sequestration. In addition, direct removal of the segment would have further compromised alveolar ridge continuity and increased the difficulty of subsequent prosthetic or reconstructive rehabilitation. Therefore, preservation of the segment was attempted, and palatal transmucosal screw fixation with long titanium screws was selected to provide necessary stability while minimizing further disruption of the residual labial soft-tissue attachment.

The operation was performed under general anesthesia. Thorough debridement of the oral and maxillofacial wounds was performed, and broken teeth, free bone fragments, and foreign bodies were removed. Exploration confirmed fractures of the left maxilla and a markedly detached dentoalveolar segment in the region of teeth 23–25. A fracture of the right mandible was also identified, with tooth 46 located within the fracture line and showing marked mobility. After extraction of tooth 46, the mandibular fracture was reduced and fixed with titanium plates and screws. The left maxillary fracture was then reduced and fixed with titanium plates and screws.

For the detached left maxillary dentoalveolar segment, careful closed reduction and anatomical assessment were first performed while preserving the residual labial soft-tissue attachment as much as possible. Because further palatal or labial flap elevation was considered likely to compromise the residual blood supply, no additional mucoperiosteal flap was raised around the segment. Instead, transmucosal fixation was performed from the palatal side. Using a 1.6-mm drill bit, drill holes were created through the palatal mucosa perpendicular to the detached fragment along the cortical surface and through the alveolar process. The reduced segment was then stabilized with two 2.0-mm self-tapping titanium screws, each 16 mm in length. After fixation, the palatal and buccal mucosa were carefully closed. Intraoperative stability was assessed based on restoration of segmental anatomical alignment, maintenance of the occlusal relationship, and absence of gross mobility on gentle manual testing after fixation. After fixation, the buccal and palatal mucosa were closed carefully. Debridement and suturing of the upper lip, lower lip, tongue, and scalp wounds were subsequently completed. At the end of the operation, the patient was transferred intubated to the intensive care unit for further monitoring. Postoperative management included routine anti-infective treatment, analgesic care, and close monitoring of wound healing. After extubation and stabilization of the general condition, the patient was instructed to maintain meticulous oral hygiene with chlorhexidine mouth rinses and gentle cleaning around the transmucosal fixation area. A liquid or soft diet was recommended during the early healing period, and direct trauma or excessive loading on the preserved dentoalveolar segment was avoided. Follow-up examinations focused on wound healing, occlusion, segment stability, tooth mobility, infection, soft-tissue dehiscence, and exposure or loosening of fixation materials.

**Figure 4:**
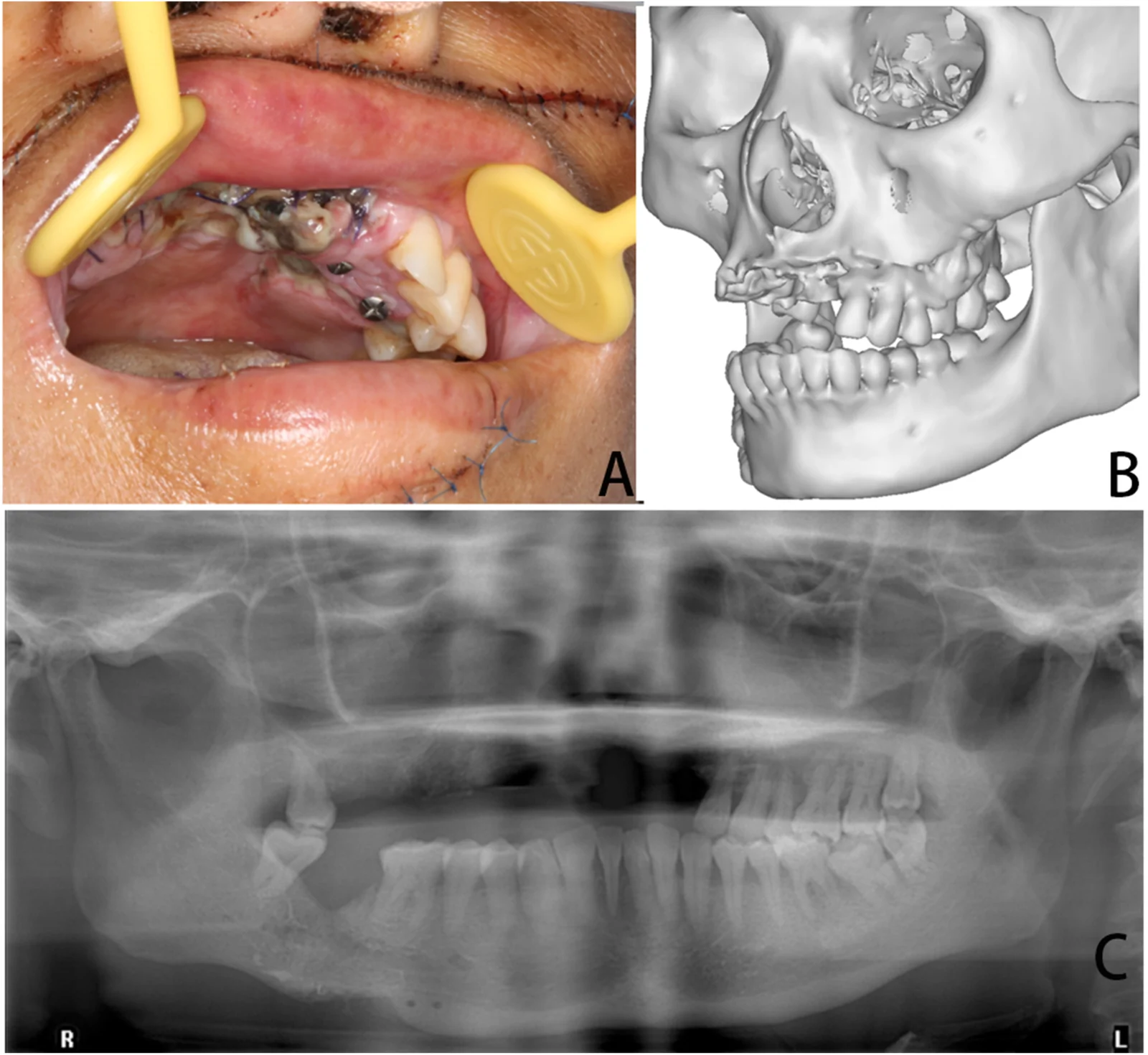
Postoperative and follow-up images of case 2. **(A)** Follow-up intraoral photograph. **(B)** Postoperative three-dimensional CT reconstruction. **(C)** Six-month panoramic radiograph.

The patient recovered uneventfully after surgery. During follow-up, the local wounds healed well, without obvious infection, soft-tissue dehiscence, or exposure of fixation materials (Figure 4A). Postoperative three-dimensional CT reconstruction demonstrated satisfactory reduction and fixation of the maxillofacial fractures (Figure 4B).

Four months after the initial surgery, removal of the titanium plates and screws was performed. Intraoperative examination showed that the previously detached dentoalveolar segment in the region of teeth 23–25 remained viable and achieved bony healing. The associated teeth were preserved *in situ* and showed no obvious mobility. A panoramic radiograph obtained 6 months after surgery further confirmed stability of the preserved teeth and restoration of local bony continuity (Figure 4C).

However, pulp vitality testing of teeth 23–25 was not systematically performed during follow-up. Therefore, although the teeth remained clinically stable and were preserved *in situ*, the pulpal status of the involved teeth could not be definitively determined. A concise timeline summarizing the major clinical events of both cases is provided in Supplementary Table 1.

## Discussion

Displaced segmental maxillary fractures involving partially detached bone segments represent a challenging subset of midfacial trauma because clinical management often requires consideration of both mechanical stability and potential segment viability (7, 14). For most displaced maxillary fractures, open reduction and internal fixation under direct visualization provides reliable stabilization. However, when a displaced segment retains only limited mucosal, periosteal, or adjacent soft-tissue attachment, extensive flap elevation may further compromise the residual blood supply and increase the risk of bone resorption, sequestration, infection, delayed union, or segmental necrosis (3, 15).

The present cases suggest that, in selected situations, fixation planning may need to consider not only sufficient stabilization but also preservation of the remaining soft-tissue envelope. Several previous reports have described mucosa-related fixation techniques with a similar rationale, although the fixation devices and clinical indications vary. A summary of representative mucosa-related fixation strategies and their relevance to the present cases is shown in Table 1.

**Table 1:** Summary of mucosa-related and minimally invasive fixation strategies.

Author	Year	No. of cases	Age	Fracture type	Fixation method	Follow-up/outcome
Cienfuegos et al. (11)	2010	45	4–56 years	Palatal fractures	Locking plates over the palatal mucosa	All healed by 12 weeks; no mucosal necrosis, fistula, or infection
Nyárády et al. (12)	2010	6	28–71 years	Alveolar process fractures	Transgingival lag-screw fixation	Uneventful healing; no bone or tooth loss
Shinohara et al. (16)	2010	1	27 years	Atypical maxillary dentoalveolar fracture	Transmucosal screw fixation	Screws removed after 5 weeks; prosthetic rehabilitation planned
Wood et al. (13)	2011	8	NR	Edentulous mandibular fractures	Transmucosal intraoral locking plate fixation	Rigid union in all patients; no postoperative infection
Benech et al. (10)	2013	11	72–86 years	Atrophic edentulous mandibular fractures	Intraoral extra-mucosal plate fixation	Most cases achieved consolidation; one infection required secondary fixation
Cortese et al. (7)	2014	1	38 years	Maxillary and alveolar fracture	Intraoral epimucosal plate fixation	Uneventful healing; hardware removed after 40 days
Sharma et al. (9)	2019	20	7–62 years	Maxillary and mandibular alveolar fractures	Transgingival lag-screw fixation	Stable anatomical reduction; no bone loss, tooth loss, or infection
Zhou et al. (17)	2020	22	25–58 years	Segmental alveolar fractures	Titanium plate or screw fixation	All fractures healed; pulp necrosis occurred in 15.6% of involved teeth; no tooth loss was observed.

NR, not reported in the accessible abstract or article summary.

As shown in Table 1, mucosa-related fixation should not be regarded as a single standardized technique. Rather, it represents a group of individualized fixation methods, including epimucosal plates, transmucosal plates, transgingival screws, and extra-mucosal screw fixation, that may be selected according to fracture pattern, segment size, soft-tissue condition, and stability requirements.

An additional practical consideration is postoperative oral hygiene. Because epimucosal and transmucosal fixation devices remain exposed within the oral cavity, they may increase plaque retention and the risk of local mucosal inflammation. Therefore, meticulous oral hygiene instruction and close follow-up are important parts of management. In the present cases, patients were advised to use regular chlorhexidine rinses, clean carefully around the exposed hardware, avoid direct trauma to the fixation area, and return for close monitoring of plaque accumulation, mucosal irritation, wound dehiscence, or hardware exposure.

In Case 1, the displaced anterior maxillary segment was relatively large and was associated with comminution and loss of local buttress support. The main technical problem was therefore not only preservation of viability, but also the need for broader stabilization across a structurally compromised anterior maxillary region. Conventional miniplate fixation under limited exposure was unlikely to provide sufficient support, whereas wider exposure for direct bone-surface fixation would have required additional stripping of the remaining soft-tissue attachments (7, 11). For this reason, intraoral epimucosal reconstruction plate fixation was selected. This approach allowed bridging stabilization of the large displaced segment while avoiding direct subperiosteal exposure of the residual soft-tissue-bearing surfaces. Its rationale is conceptually similar to previously described epimucosal or mucosa-surface plate fixation techniques for selected maxillary and palatal fractures. It should also be noted that surgery in Case 1 was performed 10 days after injury. This delay may have increased the difficulty of reduction because of early soft-tissue contracture and initial healing changes, while also reinforcing the importance of avoiding further disruption of the already compromised soft-tissue envelope.

In Case 2, the injury pattern was different. The detached segment was mainly a dentoalveolar fragment involving teeth 23, 24, and 25. Intraoperatively, this segment showed complete loss of bony continuity with the surrounding maxilla, with only limited labial mucosal attachment remaining. Therefore, palatal transmucosal screw fixation with long titanium screws was performed. This technique provided direct stabilization of the dentoalveolar segment from the palatal side while preserving the labial mucosal attachment. Similar screw-based approaches have been reported for alveolar process and dentoalveolar fractures, especially when conventional splinting is difficult or preservation of the mucoperiosteal pedicle is critical (9, 12, 13). In the present case, the segment survived, achieved bony healing, and the associated teeth were preserved *in situ* during follow-up.

The present cases highlight the possibility that a displaced or nearly detached maxillary bone segment is not necessarily nonviable in all circumstances. However, attempted preservation should be limited to carefully selected patients. In the present cases, several features supported preservation of the detached segments: the presence of residual mucosal or soft-tissue attachment suggesting potential residual vascularity; the absence of obvious segmental necrosis; the possibility of adequate debridement; the ability to reduce the segment to an acceptable anatomical and occlusal position; and the feasibility of achieving stable fixation without extensive additional mucoperiosteal stripping. In addition, preservation was considered clinically meaningful because the detached segments contributed to facial contour, alveolar ridge continuity, occlusion, and future prosthetic or reconstructive rehabilitation.

By contrast, removal of the fragment would be preferred when the detached segment appears completely devascularized or nonviable, when the bone is grossly contaminated, necrotic, infected, or severely crushed, when adequate debridement cannot be achieved, or when preservation of the segment would increase the risk of infection, sequestration, or delayed reconstruction. Fragment removal should also be considered when the segment cannot be reduced to an acceptable position or cannot be stabilized reliably under limited exposure (1, 16).

Therefore, potential contraindications to this soft-tissue-preserving fixation approach include complete loss of soft-tissue attachment, apparent devascularization, established infection or necrosis, gross contamination, non-reducible displacement, extensive comminution precluding stable fixation, and inadequate soft-tissue coverage. Because there is no universal quantitative threshold for the amount of residual soft-tissue attachment required, intraoperative judgment remains essential. The remaining attachment should appear clinically meaningful and potentially capable of maintaining at least some residual vascularity, and it should be preservable during fixation.

This report has several limitations. First, as a two-case series, its findings are inherently limited in generalizability and should be regarded as preliminary, hypothesis-generating clinical observations rather than evidence supporting a specific fixation technique. Second, the two cases differed in fracture pattern, associated injuries, timing of intervention, and fixation strategy, precluding any direct comparison between the two methods. Third, vascularity of the preserved segments was not assessed directly, and the biological basis for segment survival therefore remains inferential. In addition, follow-up was limited and non-uniform, and longer-term outcomes such as late bone resorption, periodontal status, and pulp vitality were not fully evaluated. In particular, pulp vitality testing of teeth 23–25 in Case 2 was not performed during follow-up. This limitation is clinically relevant because clinical stability of the preserved dentoalveolar segment and involved teeth does not necessarily confirm preservation of pulp vitality. Future reports should include standardized pulp vitality testing and periodontal assessment during follow-up to better evaluate tooth-related outcomes after segment preservation. Finally, selection bias is unavoidable because segment preservation was attempted only when residual soft-tissue attachment was present and reduction was considered feasible. Therefore, the main value of this report is to illustrate a biologically oriented treatment concept for selected displaced segmental maxillary fractures rather than to establish a standard treatment protocol. Further studies with larger cohorts, longer follow-up, and more objective assessment of segment and tooth viability are needed.

In summary, these two cases suggest that preservation of residual soft-tissue attachments may help maintain the viability of partially detached maxillary segments when reduction and stable fixation can be achieved without extensive additional dissection. Intraoral epimucosal reconstruction plate fixation and palatal transmucosal screw fixation were used successfully in the present patients, but these techniques should be viewed as individualized options rather than standardized treatment recommendations. They may be considered in carefully selected patients with potentially viable segments, adequate soft-tissue attachment, and achievable stability. Larger clinical series with longer follow-up are required to validate these observations and better define the indications, limitations, and long-term outcomes.

## Patient perspective

Both patients reported improvement in facial appearance, occlusal function, and chewing ability after treatment. The first patient was satisfied with the improvement in facial contour and masticatory function. The second patient expressed relief that the injured dentoalveolar segment and associated teeth were preserved. Both patients agreed to the publication of their clinical information and images.

## Data Availability

The raw data supporting the conclusions of this article will be made available by the authors, without undue reservation.
