# Supplementary material for: Case Report: Soft-tissue-preserving fixation for displaced segmental maxillary fractures involving partially detached bone segments
Source: Front Oral Health. 2026 Aug 18;7:1889166. doi: 10.3389/froh.2026.1889166 (PMC13530897; doi:10.3389/froh.2026.1889166)
Supplement: Supplementary file 1 [file Table1.docx]

Supplementary Table 1. Timeline of clinical management and follow-up

| **Time point** | **Case 1** | **Case 2** |
| --- | --- | --- |
| Injury and admission | Blunt maxillofacial injury; admitted 10 days after injury. | Open maxillofacial injury caused by an unmanned aerial vehicle propeller; admitted more than 5 hours after injury. |
| Key fracture feature | Partially detached and markedly displaced anterior maxillary segment. | Dentoalveolar segment involving teeth 23–25 with complete loss of bony continuity and limited labial mucosal attachment. |
| Initial surgery | Intraoral epimucosal reconstruction plate fixation. | Palatal transmucosal screw fixation with two long titanium screws. |
| Early postoperative course | Elastic traction was applied; postoperative CT showed satisfactory reduction and stable fixation. | The patient was transferred to the intensive care unit for monitoring; postoperative CT showed satisfactory reduction and fixation. |
| Fixation removal | Reconstruction plate removed 4 months after initial surgery. | Titanium plates and screws removed 4 months after initial surgery. |
| Additional procedure | Bone graft material and membrane were placed in the alveolar defect region of teeth 21 and 22, and autogenous bone from the left mandibular body was grafted into the region of teeth 24 and 25. | No bone grafting was performed for the preserved dentoalveolar segment. |
| Follow-up outcome | Three months after plate removal and bone grafting, the previously detached segment survived without obvious necrosis or sequestration. | At 6 months, panoramic radiography confirmed stability of the preserved teeth and restoration of local bony continuity. Pulp vitality was not systematically assessed. |
